# Prevalence of *Anaplasmataceae* and *Filariidae* species in unowned and military dogs in New Caledonia

**DOI:** 10.1002/vms3.97

**Published:** 2018-03-08

**Authors:** Mustapha Dahmani, Djamel Tahir, Olivier Cabre, Didier Raoult, Florence Fenollar, Bernard Davoust, Oleg Mediannikov

**Affiliations:** ^1^ Aix Marseille Univ CNRS IRD AP‐HM MEPHI IHU ‐ Méditerranée Infection Marseille France; ^2^ Animal Epidemiology Working Group of the Military Health Service Toulon France

**Keywords:** Dogs, kennels, New Caledonia, *Anaplasmataceae*, *Filariidae*

## Abstract

Dogs are competent reservoir hosts of several zoonotic agents, including *Filariidae* nematodes and *Anaplasmataceae* family bacteria. The latter family unites human and veterinary pathogens (*Anaplasma, Ehrlichia* and *Neorickettsia* bacteria) with *Wolbachia*, some of which are obligatory endosymbionts of pathogenic filarial nematodes. The epidemiology of *Anaplasmataceae* and *Filariidae* species infecting dogs living in kennels in New Caledonia was studied. 64 EDTA blood samples were screened for the presence of *Anaplasmataceae* and filarial nematodes. Molecular study was conducted using primers and probe targeting the of 23S rRNA long fragment of *Anaplasmataceae* species. Next, all blood sample was screened for the presence of *Filariidae* species targeting the primers and probe targeting the COI gene, as well as primers targeting the COI and 5S rRNA genes of all filarial worms. *Anaplasma platys* was identified in 8/64 (12.5, 95% confidence interval [CI]: 4.4–20.6%) and *Wolbachia* endosymbiont of *Dirofilaria immitis* in 8/64 (12.5%, CI: 4.4–20.6%). *Filariidae* species investigation was performed and showed that 11/64 (17.2%, CI: 7.9–26.4%) dogs were infected with *D*. *immitis*, whereas, 2/64 (3.1%, CI: 0.0–7.3%) were infected with *Acanthocheilonema reconditum*. Finally, we checked the occurrence of co‐infection between *Anaplasmataceae* and *Filariidae* species. Co‐occurrence with *Wolbachia* endosymbiont of *D*. *immitis* was observed in seven dogs, one dog was co‐infected with *A*. *platys* and *A*. *reconditum* and another was co‐infected with *Wolbachia* endosymbiont of *D*. *immitis* and *A*. *reconditum*. These results are the first report of *Anaplasmataceae* and *Filariidae* occurring in dogs in New Caledonia.

## Introduction

The *Anaplasmataceae* family of bacteria includes several dog‐associated pathogens such as *Anaplasma phagocytophilum, A. platys* and *Ehrlichia canis*,* E. chaffeensis, E. ewingii* and *Neorickettsia helminthoeca* (Dumler *et al*. 2001). Canine granulocytic anaplasmosis due to *A. phagocytophilum* is reported worldwide and transmitted by *Ixodes ricinus* complex ticks in Europe and North America (Dumler *et al*. 2005; Kohn *et al*. 2011). *Anaplasma phagocytophilum* and *E. canis* are the causative agents of cyclic thrombocytopenia and canine monocyte tropic ehrlichiosis, and are reported worldwide. Infections are often found in geographical regions associated with the *Rhipicephalus sanguineus s.l*. tick (Otranto *et al*. 2009; Harrus & Waner 2011). *Ehrlichia chaffeensis* and *E. ewingii* are mainly reported from the United States and primarily transmitted by *Amblyomma americanum* ticks (Yabsley *et al*. 2011; Stoffel *et al*. 2014). *Neorickettsia helminthoeca*, the agent of salmon poisoning disease, is reported on the American continents and may infect dogs who ingest parasitized salmon (Headley *et al*. 2011). Lastly, the genera of *Wolbachia* is an obligate intracellular endosymbiont and likely mutualist living within numerous arthropod species and *Filariidae* nematodes (Taylor *et al*. 2012). The detection of *Wolbachia* species in blood of dog was associated with the presence of accompanying nematodes like *D. immitis* (Mehlhorn 2008).

In the management of canine vector‐borne diseases, the diagnosis should rely on the dog's clinical status as well as epidemiological information (Otranto *et al*. 2009). New Caledonia is an archipelago of Oceania, located in Melanesia (Pacific Ocean). The aim of this paper was to get an overview of the *Anaplasmataceae* and *Filariidae* species infecting dogs on this island and the association between *Wolbachia* spp. and *Filariidae* occurrence in dog blood samples. In this paper, we report the first description of *A. platys, Wolbachia* sp. endosymbiont of *D. immitis* associated with the presence of the nematode *D. immitis*, as well as *Acanthocheilonema reconditum* from the blood of dogs in New Caledonia.

## Materials and methods

### Sampling

In April 2009, blood from dogs living in kennels in New Caledonia was sampled in EDTA‐containing tubes by cephalic venipuncture. The kennels were at the Nouméa municipal animal shelter in the district of Ducos (South Province) and the Society for the Prevention of Cruelty to Animals (SPCA) in the district of Dumbéa. Military dogs were also sampled in Nandaïl, Népoui and Tontouta. Blood‐sucking parasites found on each sampled dog were removed. The animals were examined and sampled by a veterinarian after obtaining a verbal consent from people (owners) responsible of dogs. The dogs living in kennels were either stray dogs recovered by the relevant authorities or dogs abandoned/surrendered by their owners. No data are available about sanitary status or possible prophylaxis. After transport to the laboratory in Marseille, all samples were stored at −80°C.

### Blood‐sucking parasite identification

Morphological identification was performed with a binocular microscope and carried out by an entomologist with a broad experience of arthropods of medical and veterinary importance. Blood‐sucking parasites were classified by family, genus and species using the available taxonomic keys and morphometric tables (Beaucournu & Launay 1990; Walker *et al*. 2000; Wall & Shearer 2008).

### DNA extraction and PCR amplification

Total DNA was extracted from 200 *μ*L of blood after digestion with proteinase K at +56°C for 16 h. DNA extraction was performed on the BioRobot EZ1 (Qiagen, Qiagen, Courtaboeuf, France) using a commercial DNA extraction kit (QIAamp DNA Mini Kit, Qiagen, Courtaboeuf, France) according to the manufacturer's instructions. PCR screening and amplification of *Anaplasmataceae* species was conducted using primers and probes targeting the 23S rRNA gene followed by amplification and sequencing a 485‐base pair (bp) of the same gene as previously reported (Dahmani *et al*. 2017). For *Filariidae* species investigation, all DNA samples were firstly screened by a set of primers targeting the 421 bp‐long fragment of the mitochondrial 5S rRNA gene (Mourembou *et al*. 2015) and the 1551 bp‐long fragment of the mitochondrial cytochrome oxidase subunit (COI) gene using the set of primers specifically designed to amplify most filarial DNA. All probes and primers are listed in Table 1. All samples were then screened for the presence of *D. immitis* and *Dirofilaria repens* using the specific duplex qPCR as reported previously (Tahir *et al*. 2017).

**Table 1:** Primers and probes used in this study

Targeted microorganisms	Targeted sequences	Primers and probe	Sequences 5′‐3′	Annealing temperature	References
qPCR					
*Anaplasmataceae*	23S rRNA gene	TtAna‐F	ATAAGCTGCGGGGAATTGTC	60°C	Dahmani *et al*. (2015a,2015b)
		TtAna‐R	GTAACAGGTTCGGTCCTCCA		
		TtAna‐S	FAM‐TGCAAAAGGTACGCTGTCAC‐TAMRA		
Conventional PCR					
*Anaplasmataceae*	23S rRNA gene	Ana23S‐212f	GTTGAAAARACTGATGGTATGCA	55°C	Dahmani *et al*. (2015a,2015b)
		Ana23S‐753r	TGCAAAAGGTACGCTGTCAC		Mourembou *et al*. (2015)
*Filariidae*	5S	S2	GTTAAGCAACGTTGGGCCTGG		
		S16	TTGACAGATCGGACGAGATG		
	COI (PCR)	Filcox1‐F1	TCCWGARATRGCGTTTCCTC	48°C	This study
		Filcox1‐r1	AACCATAGCCAACGCGACGAT		
	COI (Sequencing)	Filcox1‐F1	TCCWGARATRGCGTTTCCTC		
		Filcox1‐r1	AACCATAGCCAACGCGACGAT		
		Filcox1‐Fn	TTTTTGGACATCCTGARGTTT		
		Oncox‐r1	AATGAAAATGAGCYACAACAT		
		Filcox1‐rn	ACCYTGTAWTCCAGCTAAAT		

### Sequencing and phylogenetic analyses

The amplicons were sequenced on a Biosystems 3130xl Genetic Analyzer (Thermo Fisher Scientific, France) using the DNA sequencing BigDye Terminator Kit (Perkin‐Elmer, Waltham, MA, USA) as described by the manufacturer. The sequences were assembled using the ChromasPro 1.7 (Technelysium Pty Ltd., Tewantin, Australia) and compared with *Anaplasmataceae* and *Filariidae* sequences available in the GenBank database using BLAST. Sequences obtained in this study were aligned with other ticks or *Anaplasmataceae* species sequences available on GenBank using CLUSTALW on Bioedit v3 (Hall 1999), and gaps and missing data were eliminated. Phylogenetic and molecular evolutionary analysis was inferred using the maximum likelihood method used on MEGA7 (Kumar *et al*. 2016), with the complete deletion option, based on the Hasegawa–Kishino–Yano (HYK) model for nucleotide sequences. A discrete gamma distribution was used to model evolutionary rate differences among sites. Initial trees for the heuristic search were automatically obtained by applying the Neighbour‐Joining and BioNJ algorithms to a matrix of pairwise distances estimated using the Maximum Composite Likelihood (MCL) approach. Statistical support for internal branches of the trees was evaluated by bootstrapping with 1000 iterations.

## Results

A total of 64 dogs were sampled: 45 males and 19 females. Ages were available for 54 dogs. The average age was 4 years old [3 month–12 years]. In total, 51/64 (79.6, 95% CI: 69.8–89.5%) dogs were found to be infected by blood‐sucking parasites. In 44/64 (68.7–95% CI: 57.3–80.1%) dogs, 258 ticks were collected and identified as *Rhipicephalus sanguineus s.l*. Between four and eight ticks were removed from each dog. In addition, four *Ctenocephalides felis* fleas were removed from 4/64 (6.3, 95% CI: 0.3–18.1%) dogs and 24 lice *Trichodectes canis* were removed from 24/64 (37.5, 95% CI: 25.6–49.3%) dogs.

**Figure 1:**
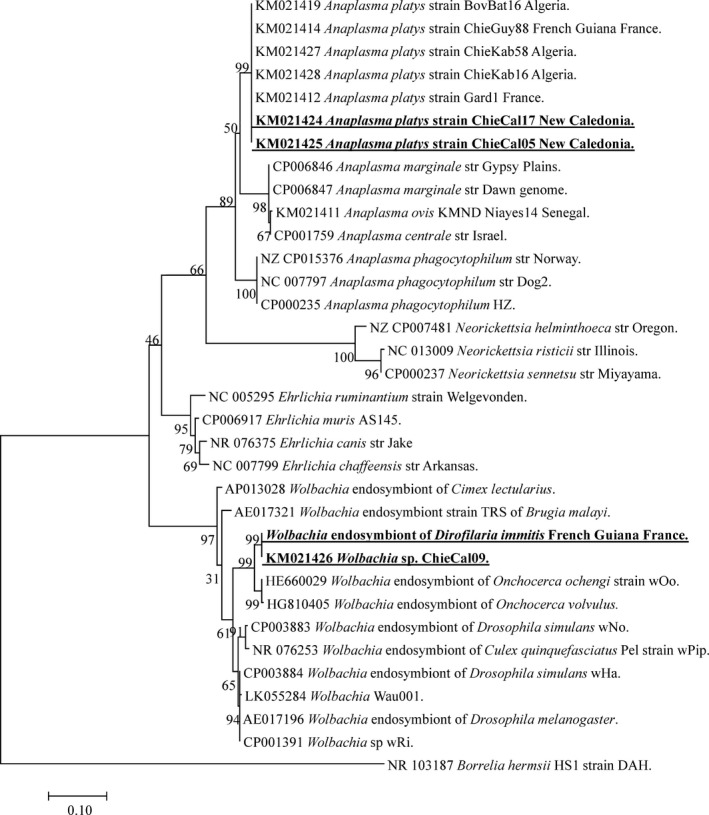
Phylogenetic tree showing the position of *Anaplasma platys* and *Wolbachia* sp. amplified from dog blood samples compared to other *Anaplasmataceae* bacteria available from GenBank. The evolutionary history was inferred using the maximum likelihood method based on the Hasegawa–Kishino–Yano model. A discrete Gamma distribution was used to model evolutionary rate differences among sites [4 categories (+G, parameter = 0.1479)]. The analysis involved 39 nucleotide sequences. All positions containing gaps and missing data were eliminated. There was a total of 434 positions in the final data set.

Screening using qPCR showed that 16/64 (25, 95% CI: 14.3–35.6%) samples contained DNA of bacteria belonging to the *Anaplasmataceae* family. After sequencing the 485 bp‐long amplicons of the 23S rRNA gene portion, BLAST analysis was conclusive for *A. platys* in eight samples, and *Wolbachia* sp. in eight samples. Two sequences of *A. platys* were obtained, respectively, from 6 to 2, then named, respectively, *A. platys* var1 and var2 (Fig. 1). The sequences of *A. platys* var1 are a 100% identity match with the *A. platys* amplified from dog blood reported in France (KM021412), Algeria (KM021427) and French Guiana (KM021414) (Dahmani *et al*. 2015a,2015b). *Anaplasma platys* var2 shared 99% identity with the *A. platys* var1 sequences cited above, and 100% homology with *A. platys* amplified from dogs in Algeria (KM021428) (Dahmani *et al*. 2015a,2015b). The total incidence was 12.5% (8/64, 95% CI: 4.4–20.6%). All infected dog were from a public (non‐military) shelter in Ducos, the incidence in this shelter was 8/25 (32, 95% CI: 13.7–50.2%).

The 23S RNA gene sequences of *Wolbachia* were identical each other and show 96% homology with *Wolbachia* endosymbionts of *Onchocerca ochengi* when BLASTed. The 23S rRNA gene sequence of *Wolbachia* from *D. immitis* was not available in GenBank, so we have sequenced it from adult filaria obtained from a dog from French Guiana. When comparing the 23S sequences of *Wolbachia* from eight dogs from New Caledonia and adult *D. immitis*, we found a 100% identity match (Fig. 1).

**Figure 2:**
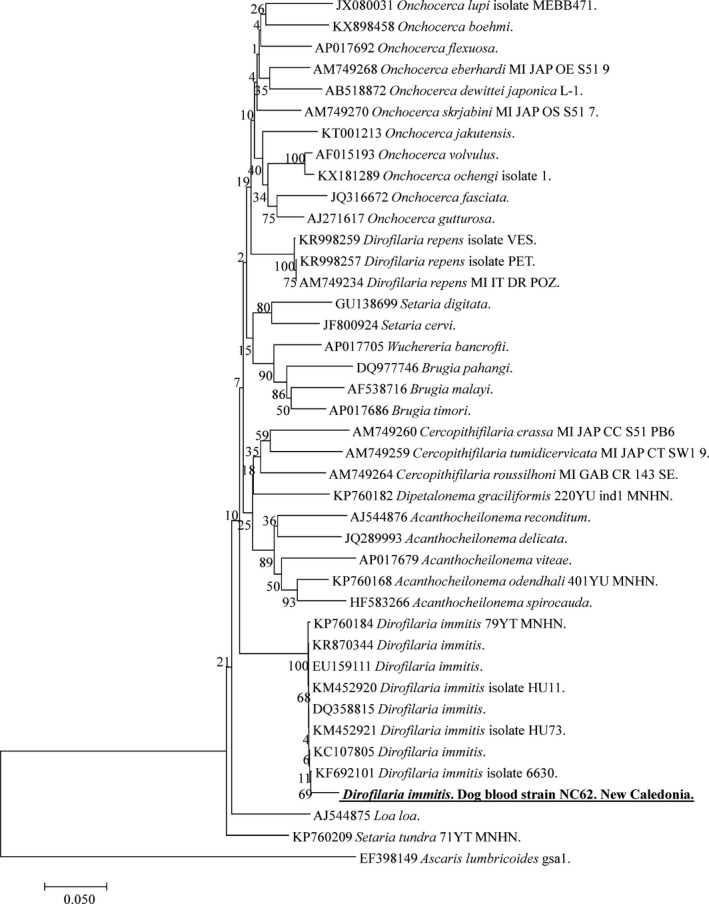
Phylogenetic tree showing the position of *Dirofilaria immitis* amplified from dog blood samples compared to other *Filariidae* nematodes available from GenBank. The evolutionary history was inferred using the maximum likelihood method based on the Hasegawa–Kishino–Yano model. A discrete Gamma distribution was used to model evolutionary rate differences among sites [4 categories (+G, parameter = 0.1479)]. The analysis involved 41 nucleotide sequences. All positions containing gaps and missing data were eliminated. There was a total of 526 positions in the final dataset.

The qPCRs specific to *D. immitis* and *D*. *repens* have identified 11/65 positive samples for only *D. immitis*. We obtained 480–583 bp‐long amplicons of filarial COI genes from nine of eleven dogs that tested positive for *D. immitis* by qPCR. The sequences obtained were identical to each to other and BLAST analysis showed 98% identity with the COI sequences of *D. immitis* reported worldwide (Fig. 2).

**Figure 3:**
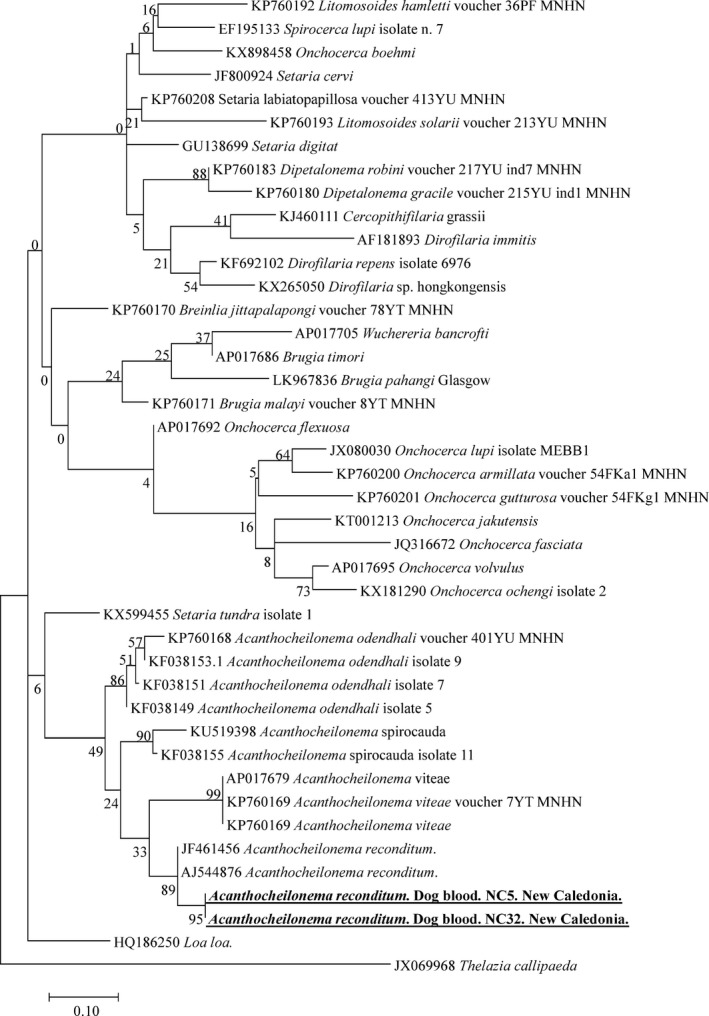
Phylogenetic tree showing the position of *Acanthocheilonema reconditum* amplified from dog blood samples compared to other *Filariidae* nematodes available from GenBank. The evolutionary history was inferred using the maximum likelihood method based on the Hasegawa–Kishino–Yano model. A discrete Gamma distribution was used to model evolutionary rate differences among sites [4 categories (+G, parameter = 0.2600)]. The analysis involved 42 nucleotide sequences. All positions containing gaps and missing data were eliminated. There was a total of 168 positions in the final dataset.

We also screened the remaining 54 dogs that were negative for *Dirofilaria‐*specific qPCR. In two samples, we obtained a band in standard PCR targeting filarial COIs. We sequenced the portion of the amplicon and obtained sequences of 559 bp (identical to each other) showing 98% homology with the *A. reconditum* reported in Italy (JF461456 and AJ544876) (Fig. 3). For one sample positive for *A. reconditum,* we also amplified the 641 bp‐long sequence of the filarial 5S rRNA gene. When BLASTed, it showed 97% identity with *Acanthocheilonema viteae* (U31646), because, unfortunately, the 5S sequences belonging to *A. reconditum* were not available on GenBank.

Co‐infection between *Anaplasmataceae* and *Filariidae* species were observed among the population of the dogs sampled in this study. Indeed, 7/8 samples positive for *Wolbachia* endosymbiont of *D. immitis* were infected with *D. immitis*, whereas the other four dogs infected with *D. immitis* did not reveal the presence of *Wolbachia*. One dog was found co‐infected both by *A. reconditum* and *Wolbachia* sp. which are usually associated with *D. immitis*. In addition, two cases of co‐infection were found: one of *A. platys* and *D. immitis* and another of *A. platys* and *A*. *reconditum*.

All the sequences obtained during this study were submitted to GenBank. For the *Anaplasmataceae* species: 23S rRNA gene for *A. platys* var1 and var2 as accession numbers KM021424 and KM021425, respectively. For *Wolbachia* sp. amplified from dog blood from New Caledonia, the accession number is KM021426, and for *Wolbachia* endosymbiont of *D. immitis* from French Guiana, KY347825. For the *Filariidae* species: the COI gene of *D. immitis* has the accession number KY347824, and *A. reconditum* has KY347823. For the 5S rRNA gene, the *A. reconditum* accession number is KY347822.

## Discussion

This study reports the first detection of *A. platys, D. immitis*, and associated *Wolbachia* sp. as well as *A. reconditum* in dog blood samples from New Caledonia. Our results show that 12.5% (8/64) of dogs sampled in this study were infected with *A. platys*. In the Pacific region, the prevalence of *A. platys* in Australia was reported as 51, 36.2 and 32% in free‐roaming dogs tested from several remote Aboriginal communities (Brown *et al*. 2006; Barker *et al*. 2012; Hii *et al*. 2012), which is almost identical to our result of 32% in the Ducos shelter. In our study, all sampled military dogs (12/64) were found free of any *Anaplasmataceae* or *Filariidae* infection, as well as of free blood‐sucking parasites. All infected dogs were from the Dumbea municipal animal shelter or the Society for the Prevention of Cruelty to Animals (SPCA) (in the district of Dumbéa). Interestingly, in Australia, the prevalence of *A. platys* in dogs living with owners was 3.8% (Hii *et al*. 2015). In Italy, the vector‐borne disease was found to be significantly higher in a public shelter than in private kennels (Pennisi *et al*. 2012). So, it seems that the social status of the dogs and the quality of the treats they receive has an impact on the prevalence of vector‐borne diseases. In this study, 51/64 (68.8%) of the sampled dogs were found to be infested with blood‐sucking parasites. *R. sanguineus s.l*. were removed from 44/64 (68.7%) dogs, whereas, *C. felis* and *T. canis* were removed from 4/64 (6.25%) and 24/64 (37.5%) dogs, respectively. The vectors of *A. platys* seem to be *R. sanguineus s.l*. worldwide (Chomel 2011). All dogs infected with *A. platys* were found to be infested with *R. sanguineus s.l*. It appears that *R. sanguineus s.l*. are also major potential vectors of *A. platys* in New Caledonia. Furthermore, *A. platys* has previously been amplified from *R. sanguineus s.l*. and from the lice *Heterodoxus spiniger* in Australia. The possible role of the *T. canis* lice must be investigated in future studies.

The simultaneous detection of the *Wolbachia* endosymbiont of *D. immitis* and the associated nematode has been previously reported in Portugal and Spain (Maia *et al*. 2016). In our study, *Wolbachia* DNA was detected in seven of the 11 dogs infected with *D. immitis*. *Dirofilaria immitis* infections are widespread in both tropical and temperate regions throughout the world, including Australia where the climate is suitable for mosquito vectors of the genera *Aedes*,* Culex* and *Anopheles* (Smout *et al*. 2016). None of the tested dogs had *D*. *repens* infections. This species was reported ones in Australia in a patient who had been living in Sydney for over 40 years but made annual trips to Corfu, Greece (Stringfellow *et al*. 2002).

In this study, two dogs were found to be infected with *A. reconditum*. The 5S rRNA sequences obtained have 641 bp, whereas primers used in this study are expected to amplify a fragment of 421 bp. Indeed, the 5S rRNA is known to be variable in length and sequence, and only part of this gene and the spliced leader sequence SL1 are conserved in *Brugia malayi*,* Brugia pahangi* and *D. immitis* (Sanpool *et al*. 2016). Interesting, one of two dogs infected with *A. reconditum* was co‐infected with *Wolbachia* endosymbiont of *D. immitis*, however, *D. immitis* was not detected from this sample. *Acanthocheilonema* spp. are known to not harbour *Wolbachia* species (Maia *et al*. 2016). It is possible that this dog was co‐infected with both *A. reconditum* and *D. immitis,* although specific qPCR for *D. immitis* was negative. Co‐infection with two *Filariidae* species has been previously reported, including *A. drancunculoides* in co‐infection with *D. immitis* in Portugal (Maia *et al*. 2016). This parasite is vectored by fleas (*C. canis, C. felis, Pulex irritans, P. simulans, Echidnophaga gallinae*) and lice (*Heterodoxus spiniger, Linognathus setosus*) (Otranto *et al*. 2013). In total, 4/64 (6.25%) and 24/64 (37.5%) dogs were found to be infested by *C. felis* fleas and *T. canis* lice, respectively, in this study. Both dogs infected with *A. reconditum* in our study were found to be infested by *R. sanguineous s.l* but not by fleas or lice. However, the role of *R. sanguineous s.l* in the transmission of *A. reconditum* has never been shown.

One dog was found to be co‐infected by *A. reconditum* and *A. platys*. Co‐infection between *A. platys* and *Rickettsiae* or other tick‐borne pathogens occurs frequently in dogs (Andersson *et al*. 2013). The increased co‐infection by multiple vector‐borne diseases and other agents in dogs is usually noted in endemic areas where the prevalence of both organisms is high, and has an important effect on disease expression and the ability to treat patients and animals effectively (Nicholson *et al*. 2010).

In conclusion, this study reports the first description of *A. platys*,* D. immitis* and associated *Wolbachia*, as well as *A. reconditum* in dogs sampled in New Caledonia. This study opens the way for further investigation of *Anaplasmataceae* and *Filariidae* species infecting dogs as little is known about species occurrences and potentially associated vectors.

## Source of funding

This study was supported by the AMIDEX project (n° ANR‐11‐IDEX‐0001‐02) funded by the “Investissements d'Avenir” French Government programme, managed by the French National Research Agency (ANR) and Foundation Méditerranée Infection (http://www.mediterranee-infection.com). The funders had no role in the study design, data collection, analysis, decision to publish, or preparation of the manuscript.

## Conflict of interest

All of the authors declare no conflict of interest related to this article.

## Ethics statement

The authors declare that all institutional guidelines for use of dogs sampled in this study were followed.

## Contributions

DR, BD, OM and FF designed the study; BD and OC collected the samples; MD and DT conducted the lab experiments; MD, DT and OM analysed the data; MD, BD and OM prepared the manuscript.
